# 620. A Study on the Development and Effectiveness of a Hospital-based Trauma Treatment Program for Firefighters

**DOI:** 10.1093/jbcr/irag033.434

**Published:** 2026-04-06

**Authors:** Se Hee Hwang, Mira Hur

**Affiliations:** Hangang Sacred Heart Hospital of Hallym University Medical Center, Seoul, Seoul-t'ukpyolsi; Institute of Korean Medicine, Daejeon University, Daejeon, Taejon-jikhalsi

## Abstract

**Introduction:**

Firefighters are frequently exposed to traumatic events, which increases their risk of depression, somatic symptoms, post-traumatic stress disorder (PTSD), and anxiety. However, hospital-based trauma treatment protocols specifically tailored to this occupational group have yet to be developed in South Korea.

This study aimed to develop and evaluate a structured, hospital-based trauma treatment protocol for firefighters that integrates mindfulness education, stabilization therapy, and cognitive processing therapy.

**Methods:**

The study included 30 firefighter participants who underwent psychological assessments and physiological tests before and after the intervention. The treatment protocol consisted of psychological lectures, mental stabilization programs, and cognitive processing therapy.

**Results:**

The results indicated significant improvements in the mental health of participants. The PHQ-9 (depression), PHQ-15 (somatic symptoms), and PCL-5 (PTSD) scores decreased significantly post-treatment (*p*<.05, *p*<.01, and *p*<.001, respectively). However, no significant change was observed in the ASI (anxiety) scores (*p*=.183).

**Conclusions:**

The hospital-based trauma treatment protocol was effective in reducing symptoms of depression, somatic symptoms, and PTSD among firefighters. These findings suggest that the protocol can be a valuable addition to mental health care for firefighters in South Korea, highlighting the need for tailored treatment programs for this high-risk occupational group.

**Applicability of Research to Practice:**

This study will serve as a foundation for future trauma recovery support for firefighters. And it is expected to contribute to improving their mental health and well-being.

**Funding for the study:**

N/A.

